# Evaluation and mechanism study of Pien Tze Huang against EV-A71 infection

**DOI:** 10.3389/fphar.2023.1251731

**Published:** 2023-10-26

**Authors:** Huiqiang Wang, Fenbei Chen, Shicong Wang, Yuhuan Li, Ting Liu, Yinghong Li, Hongbin Deng, Jingwen Dong, Jing Pang, Danqing Song, Dousheng Zhang, Juan Yu, Yanxiang Wang

**Affiliations:** ^1^ Beijing Key Laboratory of Antimicrobial Agents, Institute of Medicinal Biotechnology, Chinese Academy of Medical Sciences and Peking Union Medical College, Beijing, China; ^2^ Fujian Pien Tze Huang Enterprise Key Laboratory of Natural Medicine Research and Development, Zhangzhou, China; ^3^ Institute for Drug Control, National Institute for Food and Drug Control, Beijing, China

**Keywords:** enterovirus A71, Pien Tze Huang, multicomponent drug, host, cytokine, proteomics

## Abstract

Hand, foot, and mouth disease (HFMD) caused by enterovirus A71 (EV-A71) infection, currently lacks specific preventive and therapeutic interventions. Here, we demonstrated that Pien Tze Huang (PZH) could dose-dependently inhibit EV-A71 replication at the cellular level, resulting in significant reductions in EV-A71 virus protein 1 (VP1) expression and viral yields in Vero and human rhabdomyosarcoma cells. More importantly, we confirmed that PZH could protect mice from EV-A71 infection for the first time, with Ribavirin serving as a positive control. PZH treatment reduced EV-A71 VP1 protein expression, viral yields in infected muscles, and improved muscle pathology. Additionally, we conducted a preliminary mechanism study using quantitative proteomics. The results suggested that the suppression of the PI3K/AKT/mTOR and NF-κB signaling pathways may contribute to the anti-EV-A71 activity of PZH. These findings provide strong evidence supporting the potential therapeutic application of PZH for EV-A71 infection management.

## 1 Introduction

Enterovirus A71 (EV-A71) is a single-stranded RNA virus that is primarily responsible for hand, foot and mouse disease (HFMD). It predominantly affects children and is characterized by skin rash, fever, and severe neurological complications such as inflammation of central nervous system (CNS), neurogenic pulmonary oedema and even death (Solomon et al., 2010). EV-A71 belongs to the enterovirus genus of the Picornaviridae family. Its genome RNA of EV-A71 contains an open reading frame (ORF) encoding a polyprotein consisting of 2,193 amino acid residues. The polyprotein is subsequently processed by viral proteases into four structural virus proteins (VP), namely, VP1 to VP4, and seven non-structural proteins (2A, 2B, 2C, 3A, 3B, 3C, and 3D) (Wang and Li, 2019). EV-A71 was first isolated from patients in California in 1969. Many large-scale outbreaks have occurred worldwide in the following decades (Solomon et al., 2010).

Upon infection, host pathogen sensors [RIG-I-Like Receptors (RLRs), Toll-Like Receptors (TLRs), and NOD-Like Receptors (NLRs)] recognize endogenous DNA and RNA from EV-A71 and dead cells. This recognition triggers host downstream signaling such as NF-κB signaling, which in turn activates antiviral immunity and inflammatory responses. In addition, the accumulation of reactive oxygen species (ROS) after infection can activate kinases [such as Phosphoinositide 3-kinase (PI3K) and mitogen-activated protein kinases (MAPK)] and transcription factors (such as NF-κB), leading to the release of pro-inflammatory cytokines and chemokines (Duan et al., 2014; Pathinayake et al., 2015; Jin et al., 2018). The severe inflammatory response can lead to serious damage in different organs, especially in young children, highlighting the need for effective vaccines and drugs against EV-A71 (Swain et al., 2022).

Ribavirin (RBV, aerosol inhalation/oral) is currently used clinically to treat mild HFMD caused by EV-A71, but its therapeutic effect is not clear (Abzug, 2014). It is important to note that the World Health Organization (WHO) and Food and Drug Administration (FDA) have imposed strict limitations on the use of RBV in pediatric patients due to concerns regarding safety and adverse effects. As with other viral diseases, recombinant human interferon shows great potential in the short-term treatment of HFMD (Lin et al., 2019). Its use is still limited due to adverse reactions such as fever and pain. In addition, certain clinical treatments incorporate anti-inflammatory Chinese medicines as adjuncts, but their effectiveness and safety remain controversial (Li et al., 2013; Lin et al., 2016). Thus, discovering novel anti-EV-A71 candidates is urgently needed.

Pien Tze Huang (PZH, referred to as anti-inflammatory tablets in English), is a traditional Chinese herbal formula composed of four traditional Chinese medicine ingredients: *Panax notoginseng* (Burkill) F.H. Chen (85%), *Moschus* (3%), *Calculus bovis* (5%), and *Snake gall* (7%) (Huang et al., 2019). This medicine is now under the first-grade state protection, which is the highest level for Chinese patent medicine protection. Previous studies utilizing HPLC-QTOF-MS have identified the characteristic bioactive compounds in PZH, including saponins (notoginsenoside R1, ginsenoside Rb1, ginsenoside Rg1, etc.), bile acids (glycocholic acid, glycodeoxycholic acid, Cholic acid, etc.), taurine, and muscone. These compounds have demonstrated significant inhibitory effects on LPS-induced TNF-α production (Huang et al., 2016). As its name implies, PZH has historically been employed for the treatment of inflammation and general pain. It has been extensively used for liver injury and fibrosis, cerebral chronic ischemia, hypertensive stroke, ulcer, fever and various inflammation disease (Schramm, 1955; Zhang et al., 2010; Jinyan et al., 2017; Zheng et al., 2019; Huang et al., 2021; Zhu et al., 2021). Furthermore, clinical studies have investigated the efficacy of PZH in the treatment of chronic hepatitis B, aphthous ulcer, colon cancer and liver cancer (Liu and Zhou, 2006; Meng and Gu, 2008; Lim and Zhu, 2012; Zhong et al., 2017).

Recently, it is reported that PZH was effective for HFMD and herpetic angina, conditions typically caused by EV-A71. PZH has demonstrated the ability to suppress EV-A71 replication and interfere with VP1 expression within a non-cytotoxic concentration range *in vitro* (Lin et al., 2022). However, *in vivo* efficacy and mechanism of PZH against EV-A71 remain to be illustrated. In this study, the inhibitory effects of PZH on EV-A71 was investigated both *in vitro* and *in vivo*. Additionally, the mechanism of PZH antiviral activity was also investigated via quantitative proteomics.

## 2 Materials and methods

### 2.1 Medicine

The sample of PZH was provided by Fujian Pien Tze Huang Pharmaceutical Co., LTD. PZH powder (400 mg) was weighed and added to 20 mL phosphate-buffered saline (PBS), dissolved by ultrasound in a water bath at 37°C for 30 min, and placed at room temperature for 20 h. The supernatant was obtained by centrifugation, filtered and packaged with 0.22 μm filter membrane, and stored at −20°C for future use. The storage concentration was recorded as 20 mg/mL, and diluted to the required concentration.

### 2.2 Viral strains and cell lines

The human rhabdomyosarcoma (RD) cells were purchased from the Cellular Cultivation Center of Chinese Academy of Sciences (CAS). The cells were grown in Dulbecco’s modified Eagle’s medium (DMEM) supplemented with 10% fetal bovine serum (FBS; Gibco, United States) and 1% penicillin–streptomycin (Invitrogen, Carlsbad, CA, United States). Vero cells were obtained from the American Type Culture Collection (ATCC) and cultured in modified Eagle’s medium (MEM) supplemented with 10% inactivated FBS (Gibco, Grand Island, NY, United States) and 1% penicillin–streptomycin.

The EV-A71 strain H (VR-1432) was acquired from ATCC, while the mouse-adapted EV-A71 strain (EV-A71-H-MA) was generated through adaptive transmission in mice. 200 μL EV-A71-H (about 400 TCID_50_ (50% tissue culture infective doses)) was intraperitoneally injected into 12-day-old BALB/c mice, with 5–6 mice per group. After 3–5 days, the hind limb muscles were ground to obtain tissue grinding liquid, which was diluted and inoculated into Vero cells. Upon the appearance of cytopathic effects (CPE) in over 90% of the cells, the virus was collected. The EV-A71-H-M1 virus was obtained by centrifugation after repeated freezing and thawing at −80°C for three times. Then, EV-A71-H-M1 was injected into 12-day-old BALB/c mice, and the above operation was repeated six times until EV-A71-H-MA was obtained (Wang et al., 2013; Wang et al., 2019; Wang et al., 2023). All viral strains were propagated in Vero cells.

### 2.3 Mice

Specific Pathogen-Free pregnant Institute of Cancer Research (ICR) mice were sourced from SPF (Beijing) Biotechnology Co., Ltd. They were individually housed in ventilated cages to ensure proper airflow. All experiments involving the mice were carried out in accordance with animal biosafety level 2 (ABSL-2) conditions and followed the guidelines of manipulative technique for the care and use of laboratory animals in animal experimentation.

### 2.4 Cytotoxicity assay

The cytotoxic effect of PZH or RBV on cells was assayed by CPE method as described previously. Briefly, Vero or RD cells were seeded at a density of 3 × 10^4^ cells/well in 96-well culture plates and incubated overnight. Then, different concentrations of PZH or RBV were applied in triplicate. After a 3-day incubation, the concentration that inhibits 50% cellular growth in comparison with untreated control was determined as the median toxic concentration (TC_50_). The TC_50_ was calculated by Reed and Muench method (Reed and Muench, 1938).

### 2.5 CPE inhibition assay for anti-EV-A71

The anti-enteroviruses activities of PZH and RBV were evaluated using CPE assay as described previously. Briefly, Vero or RD cells were infected with 100TCID_50_ EV-A71 for 1 h at 37°C. Subsequently, either PBS or various concentrations of PZH and RBV were added to the cells for 48 h until the CPE of virus control group reached 100%. Then the CPE was recorded for the different drug treatment groups at varying concentrations. The 50% inhibitory concentration (IC_50_) was determined using the Reed and Muench method (Reed and Muench, 1938). The selectivity index (SI) was calculated as the ratio of TC_50_/IC_50_.

### 2.6 Western blot (WB) assay

WB experiments were performed as described previously (Zhong et al., 2022). Vero or RD cells were seeded at a density of 3 × 10^5^ cells/well in 6-well culture plates. After 24 h of incubation, the culture medium was removed, and the cells were either mock-infected or infected with EV-A71 (H, MOI = 0.01 (multiplicity of infection)) for 1 h. Subsequently, cells were subjected to PZH or RBV treatment for 24 h. Total proteins were extracted by M-PER Mammalian Protein Extraction Reagent (Thermo Fisher Scientific, Waltham, MA, United States) supplemented with 1% halt protease and phosphatase inhibitor single-use cocktail (Thermo Fisher Scientific, Waltham, MA, United States). Proteins were then separated by SDS-polyacrylamide gel electrophoresis and transferred onto PVDF membranes (Millipore, MA, United States). The primary antibodies against β-actin, p-AKT, AKT, p-PI3K, PI3K, p-P65, P65, p-JNK, JNK were purchased from Cell Signaling Technology (Beverly, MA, United States), EV-A71 VP1 from GeneTex (California, United States), p-mTOR and mTOR from Abcam (Cambridge, MA, United States). The goat anti-rabbit and anti-mouse HRP-labeled secondary antibodies were purchased from Cell Signaling Technology.

### 2.7 In-cell Western assay

The in-cell western assay was performed following the methods described in a previous study (Wang et al., 2023). Vero or RD cells were seeded at a density of 3 × 10^4^ cells/well in 96-well black culture plates. After 24 h of incubation, the culture medium was removed, and the cells were either mock-infected or infected with EV-A71 (H, MOI = 0.01) for 1 h. Then, cells were subjected to PZH or RBV treatment for 24 h. The cells were fixed using 4% paraformaldehyde and permeabilized with 0.5% Triton X-100 at room temperature for 15 min. Subsequently, the cells were blocked with Odyssey Blocking Buffer for 1 h at room temperature. The cells were incubated with GAPDH antibody (Proteintech) and EV-A71 VP1 antibody (GeneTex) at room temperature for 2 h and washed with PBS for three times. Then the samples were incubated with IRDye 800CW Goat anti-Mouse secondary antibody (LI-COR, United States) and IRDye 680RD Goat anti-Rabbit secondary antibody (LI-COR) and washed with PBST. Finally, the images were captured using the Odyssey CLx system (LI-COR, United States).

### 2.8 Real-time reverse transcription-PCR (qRT-PCR)

Vero or RD cells were seeded at a density of 3 × 10^5^ cells/well in 6-well culture plates. After 24 h incubation, the culture medium was removed, and the cells were mock-infected or infected with EV-A71 (H, MOI = 0.01) for 1 h. Then, cells were subjected to PZH or RBV treatment for 24 h. RNA extraction was performed using the RNeasy Mini Kit (Qiagen, United States). Subsequently, all RNA samples were analyzed using the TransScript II Green One-Step qRT-PCR SuperMix (TransGen Biotech) and the ABI 7500 Fast Real-Time PCR system (Applied Biosystems). The quantification of EV-A71 VP1 RNA levels was carried out using One-Step qRT-PCR with the following primers: VP1-forward (5′-GAT​ATC​CCA​CAT​TCG​GTG​A-3′) and VP1-reverse (5′-TAG​GAC​ACG​CTC​CAT​ACT​CAA​G-3′). To standardize the examined mRNAs, GAPDH mRNA was utilized as an internal control. The primers used for GAPDH were GAPDH-forward (5′-GAA​GGT​GAA​GGT​CGG​AGT​C-3′) and GAPDH-reverse (5′-GAA​GAT​GGT​GAT​GGG​ATT​TC-3′). The comparative fold change of the detected RNA specimens was determined using the comparative 2^−ΔΔCT^ method.

### 2.9 Virus titer determination *in vitro*

Vero or RD cells were seeded at a density of 3 × 10^5^ cells/well in 6-well culture plates. After 24 h incubation, the culture medium was removed, and the cells were either mock-infected or infected with EV-A71 (H, MOI = 0.01) for 1 h. Then, cells were subjected to PZH or RBV treatment for 24 h. After three freeze-thaw cycles of cells and culture medium, the titers of virus in the cell lysates were determined in Vero cells by a Cytopathic effect (CPE) assay. Briefly, Vero cells were seeded at a density of 3 × 10^4^ cells/well in 96-well culture plates. After 24 h of incubation, the culture medium was removed, and the cells were infected with the cell lysates in serum-free medium for 1 h at 37°C. Following the infection period, unbound viruses were removed, and MEM supplemented with 2% FBS was added for further 72-h incubation. The growth state of cells in each pore was observed by a microscope. The cells with different concentrations of the drug were compared with normal, untreated cells. They were labeled as 4+ (75%–100% cell death), 3+ (50%–75% cell death), 2+ (25%–50% cell death), 1+ (0%–25% cell death), 0+ (no changes in cell morphology or all survival), and 0+ indicates non-toxicity. The TCID_50_ was measured using the Reed and Muench method (Reed and Muench, 1938).

### 2.10 PZH protected mice against EV-A71 infection

The *in vivo* antiviral activity of PZH was assessed following the methods, which was outlined in a previous study (Wang et al., 2019; Wang et al., 2023). All animal procedures were conducted in compliance with the approved standard operating procedures of the Institutional Animal Ethics Committee at the Institute of Medicinal Biotechnology, Chinese Academy of Medical Sciences and Peking Union Medical College. For the *in vivo* assessment of mortality and morbidity, 11-day-old ICR mice were infected with EV-A71-H-MA (20000 TCID_50_) for 1 h by intraperitional inoculation. Afterword, the infected mice received intragastric administration of PZH at different doses (170, 56.7, and 18.9 mg/kg). Additionally, the mice in positive control group were intraperitoneally injected with RBV at a dose of 50 mg/kg once daily for a total of 7 days. The mice in placebo group were infected with an equivalent volume of PBS, serving as the infected control. Each group consisted of 10 mice. The symptoms and survival rates of the infected mice were monitored daily for 2 weeks. The clinical scores were assigned based on previously established standards. The scoring system graded the severity of clinical disease as follows: 0 for healthy, 1 for scattered hair, 2 for limb-shaking weakness, 3 for hind limb paralysis, and 4 for moribund or deceased animals.

### 2.11 PZH inhibits EV-A71 replication *in vivo*

For *in vivo* assessment of viral propagation and histopathology in the EV-A71-H-MA infected mice, the protocol for drugs treatment and EV-A71-H-MA infection was the same as described above. 11-Day-old ICR mice were infected with EV-A71-H-MA by intraperitional inoculation at a dose of 20000 TCID50 for 1 h. Following the initial infection, the infected mice received intragastric administration of PZH at three different doses: 170, 56.7, and 18.9 mg/kg. Additionally, the mice were intraperitoneally injected with RBV at a dose of 50 mg/kg once daily. Mice were dissected at 5 days post-infection. Muscle tissues were fixed in 10% neutral buffered formalin for subsequent hematoxylin and eosin (H&E) staining, and immunohistochemical (IHC) analyses using an antibody against EV-A71 VP1 (GeneTex, CA, United States). Muscle tissues from other mice were frozen for virus titer assays.

### 2.12 Virus titer determination in the infected mice

Virus titer determination was performed following established methods. The muscle samples were weighed and stored at −80°C. The muscles were homogenized in cold MEM using the Precellys Evolution Super Homogenizer (bertin, France). The virus titers were measured through CPE assays conducted in Vero cells. Vero cells were seeded at a density of 3 × 10^4^ cells/well in 96-well culture plates. After 24 h incubation, the culture medium was removed, and the cells were infected with the clarified homogenate supernatant in serum-free medium for 1 h at 37°C. Following the infection period, unbound viruses were removed, and MEM supplemented with 2% FBS was added for further 72-h incubation. The TCID_50_ was measured using the Reed and Muench method (Reed and Muench, 1938).

### 2.13 Proteomic analysis

RD cells were infected with EV-A71 at an MOI of 0.01 for 1 h. Following infection, the culture media was replaced with DMEM supplemented with 2% FBS and either 20 μg/mL of PZH or PBS, and the cells were incubated for 24 h. Afterward, the media was removed, and the RD cells were washed with PBS. To extract the cellular proteins, the cells were sonicated in the presence of 8 M urea. The resulting lysates were centrifuged at 12,000 g for 20 min at 4°C, and the supernatant was collected. The protein concentration was measured using the Bradford protein assay. Next, diphtheria-tetanus toxoid was added to the protein at a final concentration of 5 mM, and the mixture was incubated at 37°C for 1 h. Subsequently, iodoacetamide was added to achieve a final concentration of 10 mM and incubated in the dark at room temperature for 45 min. The samples were then diluted with four volumes of 25 mM ammonium bicarbonate solution, and trypsin was added to digest the proteins at a ratio of 1:50. The mixture was incubated overnight at 37°C. Finally, the protein was desalted using C18 columns.

The LC-MS/MS analysis was performed using a RIGOL L-3000 HPLC system coupled to a Thermo Q-Exactive HFX mass spectrometry instrument equipped with a Nanospray Flex™ (NSI) ion source. The ion source operated at 2.4 kV with a nebulizing gas flow of 275°C. A mobile phase was prepared with water and 0.1% formic acid as phase A, while phase B consisted of 80% acetone and 0.1% formic acid. The sample was dissolved in phase A and eluted from a 12 cm × 150 μm ID C18 column at a flow rate of 600 nL/min. The data-dependent acquisition mode was selected for the analysis, with a full scan range of m/z 350 to 1,500. MS1 resolution was set at 120,000 (at 200 m/z) with an automatic gain control (AGC) target value of 3 × 10^6^. The top 40 parent ions exhibiting high ionic strength were subjected to fragmentation using Higher-energy C-trap dissociation (HCD) at a collision energy of 27%. MS2 resolution was set at 15,000 (at 200 m/z) with an AGC target value of 5 × 10^4^ for MS2. The resulting data was processed using Proteome Discoverer 2.4 software, and the protein database was sourced from UniProt.

### 2.14 Bioinformatics analysis

The significantly different proteins (fold change >1.5, *p* < 0.05) were subjected to bioinformatics analysis, including Gene Ontology (GO) annotation database (http://www.geneontology.org/) and Kyoto Encyclopedia of Genes and Genomes (KEGG) pathway enrichment (http://www.genome.jp/kegg/).

### 2.15 Quantification and statistical analysis

Statistical analyses were conducted using either SigmaPlot 13 or GraphPad Prism 7.0 software. The results are presented as mean ± standard deviation (SD). To determine statistical significance, a one-way analysis of variance (ANOVA) with Holm-Sidak multiple comparisons test and t tests were employed. Survival studies were analyzed using the Log-Rank (Mantel-Cox) test with GraphPad Prism 7.0 software. The clinical score of the disease was assessed using the Ridit assay with SigmaPlot 13 software. A significance level of *p* < 0.05 was considered statistically significant.

## 3 Results

### 3.1 PZH inhibits EV-A71 replication

To evaluate the antiviral effect of PZH at the cellular level, we initially determined the TC_50_ value of PZH *in vitro*. According to Table 1, the TC_50_ value of PZH was 384.90 ± 0 μg/mL in both Vero cells and RD cells. At non-toxic concentrations, we further evaluated the inhibitory activity of PZH against EV-A71. According to Table 1, the IC_50_ value of PZH for EV-A71 H strain was 44.27 ± 7.55 μg/mL in Vero cells. Further verification was conducted using RD cells, and PZH exhibited a better inhibitory potency with an IC_50_ value of 31.92 ± 5.69 μg/mL compared to RBV. The SI was 8.69 in Vero cells and 12.06 in RD cells (Table 1). These results indicated that PZH exhibited a substantial ability to impede the replication of EV-A71 at the cellular level.

**TABLE 1:** The efficiency of PZH and RBV against EV-A71 *in vitro*.

Cells compound	Vero			RD		
	TC_50_ (μg/mL)^a^	IC_50_ (μg/mL)^a^	SI	TC_50_ (μg/mL)	IC_50_ (μg/mL)	SI
PZH	384.90 ± 0	44.27 ± 7.55	8.69	384.90 ± 0	31.92 ± 5.69	12.06
RBV	6,546.91 ± 669.79	46.15 ± 14.59	141.86	2053.40 ± 223.26	168.30 ± 24.65	12.20

^a^ Values presented in this table correspond to the mean of three independent experiments.

**FIGURE 1:**
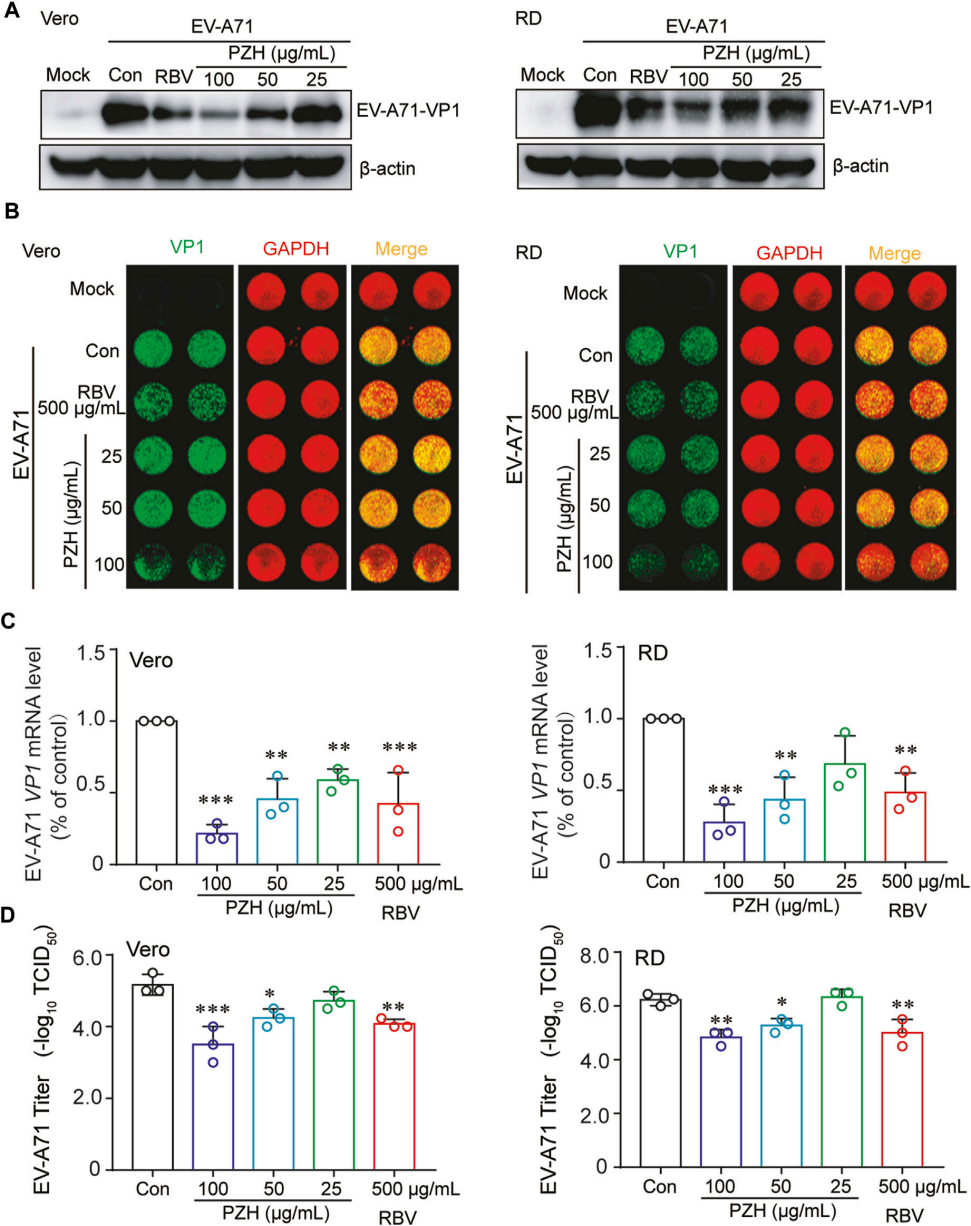
PZH inhibits EV-A71 replication. Vero and RD cells were mock-infected or infected with EV-A71 (H, MOI = 0.01) for 1 h. Then, cells were subjected to PZH or RBV treatment for 24 h. The cells were collected for WB assay using specific antibodies **(A)**, In-cell WB assay using indicated antibodies **(B)**, qRT-PCR assay **(C)** (*n* = 3) and viral titers detection by the end-point dilution assay (TCID_50_) in Vero cells **(D)** (*n* = 3). Statistical analysis was conducted using one-way ANOVA with Holm-Sidak multiple comparisons test compared to the control group and significance was indicated by *p < 0.05, **p < 0.01 and ***p < 0.001.

In order to investigate the effect of PZH on the biological synthesis of EV-A71, the EV-A71 (H strain) infected cells were treated with PZH and RBV, respectively. The expression of VP1 protein was measured by WB and In-cell WB. As depicted in Figure 1A, PZH demonstrated a dose-dependent reduction in VP1 protein expression in both Vero and RD cells. Consistent results are also observed in the In-cell WB assay (Figure 1B), corroborating the WB findings. PZH treatment also exhibited a dose-dependent decrease in EV-A71 RNA levels, as measured by RT-qPCR assay in both Vero and RD cells (Figure 1C). Furthermore, the viral yields were also decreased in both Vero and RD cells upon PZH treatment (Figure 1D). RBV treatment also exhibited inhibitory effects on VP1 protein expression (Figures 1A, B), intracellular viral RNA (Figure 1C) and the yields of EV-A71 (Figure 1D). These results demonstrated the potent anti-EV-A71 activity of PZH.

### 3.2 PZH protects mice from EV-A71 infection

**FIGURE 2:**
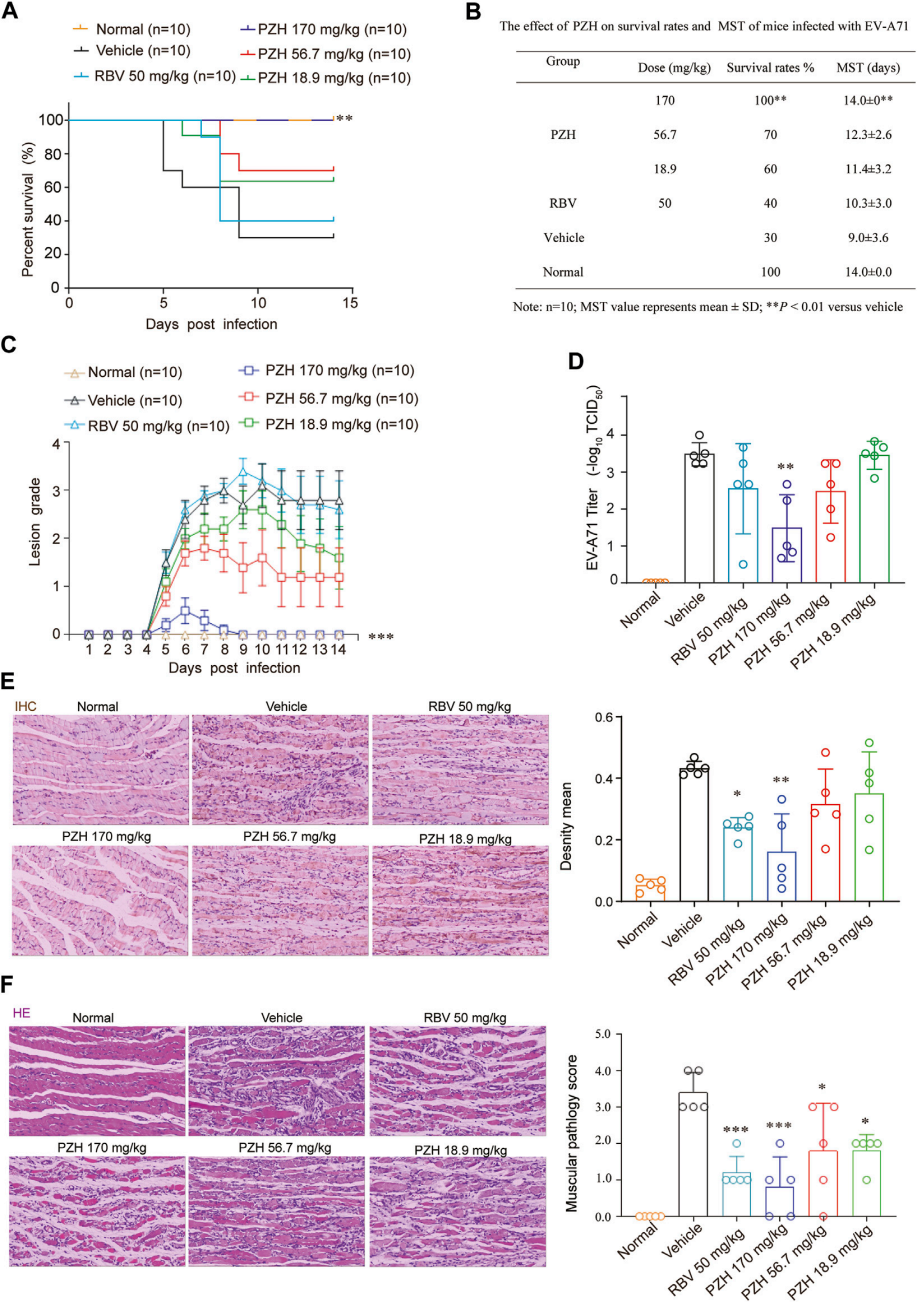
PZH protects mice from EV-A71 infection. **(A–C)** 11-Day ICR mice were intraperitoneally inoculated with EV-A71-H-MA (20000 TCID_50_) for 1 h. Following the infection, the infected mice received intragastric administration of PZH (170, 56.7 and 18.9 mg/kg) and intraperitoneal injection of RBV (50 mg/kg) daily for 7 days. The survival of the mice was monitored daily for 14 days **(A, B)** and the clinical scores were recorded for the same duration **(C)**. **p < 0.01 by A Log-Rank (Mantel-Cox) assay **(A,B)** and ***p < 0.001 by Ridit assay **(C)**. **(D–F)** The mice were dissected at day 5 post-infection. Muscle tissues were subjected to viral titer assays [**(D)**, *n* = 5]. Immunohistochemistry analyses were performed on muscle tissue sections using EV-A71 VP1 antibody [**(E)**, *n* = 5)]. Paraffin-embedded sections of muscle tissues were prepared and examined using H&E stain [**(F)**, *n* = 5]. *p < 0.05, **p < 0.01 and ***p < 0.001 by one-way ANOVA with Holm-Sidak multiple comparisons test or Ridit assay, compared to the vehicle group. *p < 0.05, **p < 0.01 and ***p < 0.001 by one-way ANOVA with Holm-Sidak multiple comparisons test **(D,E)** or Ridit assay **(F)**, compared with vehicle group.

Immature 11-day ICR mice were chose as models to assess the protective effect of against EV-A71 infection. The mice were intraperitoneally injected with EV-A71-H-MA (20000 TCID_50_) for 1 h. Subsequently, the infected mice received intragastric administration of PZH (170, 56.7 and 18.9 mg/kg) or intraperitoneal injection of RBV at a dose of 50 mg/kg. In comparison to the vehicle control, PZH treatment led to an improvement in the survival rate of mice and offered protection to the infected mice against lethal EV-A71 challenge (Figures 2A, B). The mice in vehicle control group perished due to EV-A71 infection with a mean survival time (MST) of 9.0 ± 3.6 days (Figure 4B). Administration of high-dose (170 mg/kg) PZH significantly protected the mice from mortality with an MST of 14.0 ± 0 days (Figure 2B) and increased the survival rates of infected mice. Notably, 170 mg/kg PZH treatment considerably ameliorated the clinical manifestation scores of mice exposed to EV-A71, approaching a normal grade, compared with other PZH treatment groups (Figure 2C). However, RBV treatment (50 mg/kg) through intraperitoneal injection did not provide significant protection to mice against EV-A71 infection. These results indicate that PZH could provide protection to mice against EV-A71 infection at an appropriate dosage.

**FIGURE 3:**
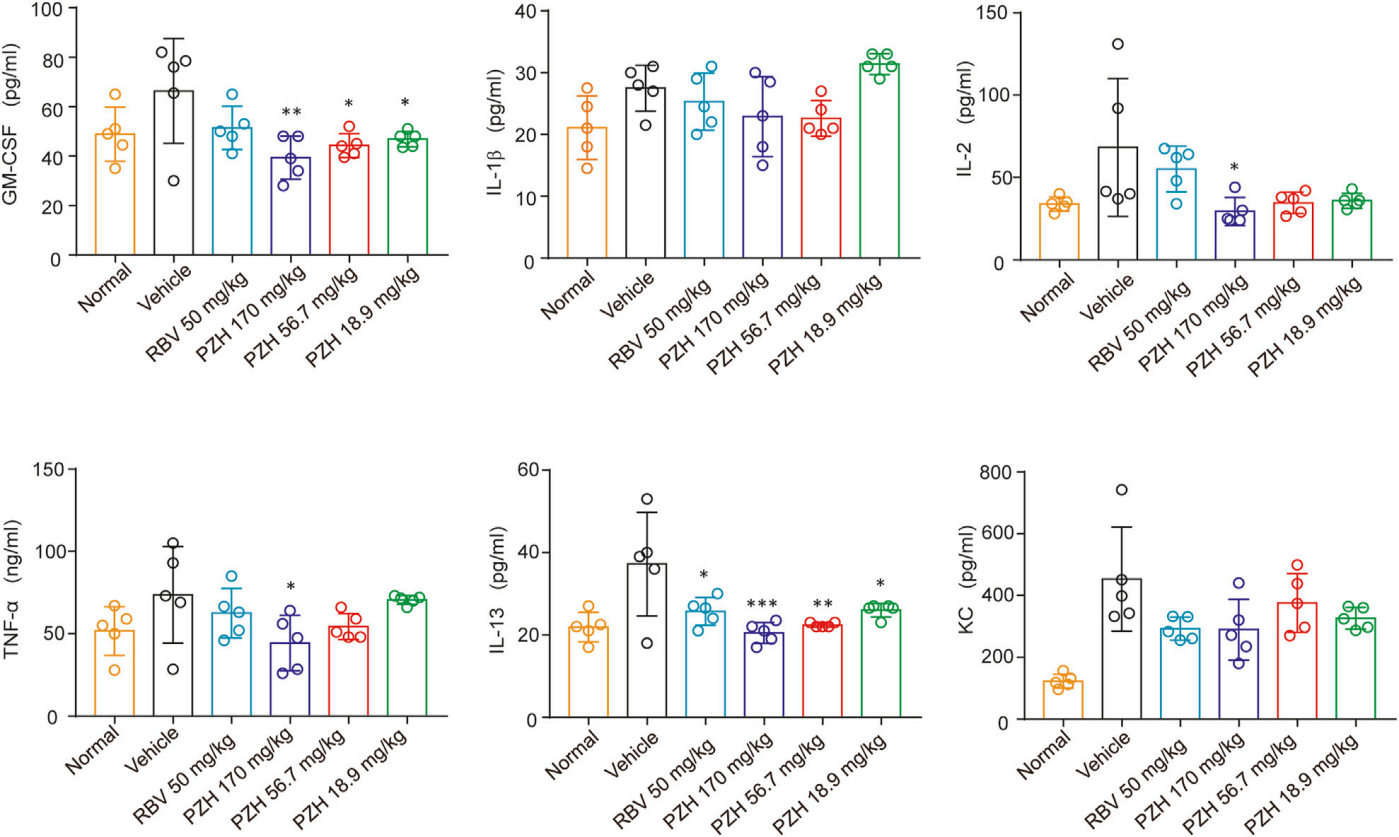
PZH downregulates cytokines in mice serum. 11-Day ICR mice were intraperitoneally inoculated with EV-A71-H-MA (20000 TCID_50_) for 1 h. Following the infection, the infected mice received intragastric administration of PZH (170, 56.7 and 18.9 mg/kg) and intraperitoneal injection of RBV (50 mg/kg). Serum from mice was collected at 5 dpi for testing. *p < 0.05, **p < 0.01, ***p < 0.001 by one-way ANOVA with Holm-Sidak multiple comparisons test, compared with vehicle group.

Due to the significantly higher levels of EV-A71 titers detected in hind-limb muscle tissues compared to other tissues (Wang et al., 2013), the expressions of EV-A71 VP1 protein and viral yields in hind limb muscle tissue were examined. The results demonstrated that PZH treatment exhibited a dose-dependent decrease in yields of EV-A71 in infected muscles (Figure 2D). And it also inhibited the expression of EV-A71 VP1 protein in hind-limb muscle tissue (Figure 2E). In line with the decrease of viral yields, PZH administration led to an improvement in muscle pathological manifestations, as evidenced by H&E staining of muscle tissue sections (Figure 2F). Considering that PZH treatment alleviates the pathological injury in mouse muscle tissue, we further examined the changes of cytokines in mice serum, with or without PZH treatments. As shown in Figure 3, PZH dose-dependently downregulated the expressions of granulocyte-macrophage colony-stimulating factor (GM-CSF), interleukin-1 beta (IL-1β), interleukin-2 (IL-2), tumour necrosis factor alpha (TNF-α), interleukin-13 (IL-13), and keratinocytes (KC). Particularly, high-dose (170 mg/kg) PZH treatment exhibited comparable or even more potent cytokine regulatory efficacy than RBV. These results indicated that PZH might exert its anti-EV-A71 activity by regulating cytokines release, which evoked us to carry out further mechanism study.

### 3.3 Quantitative proteomics analysis of EV-A71-infected RD cells after PZH treatment

**FIGURE 4:**
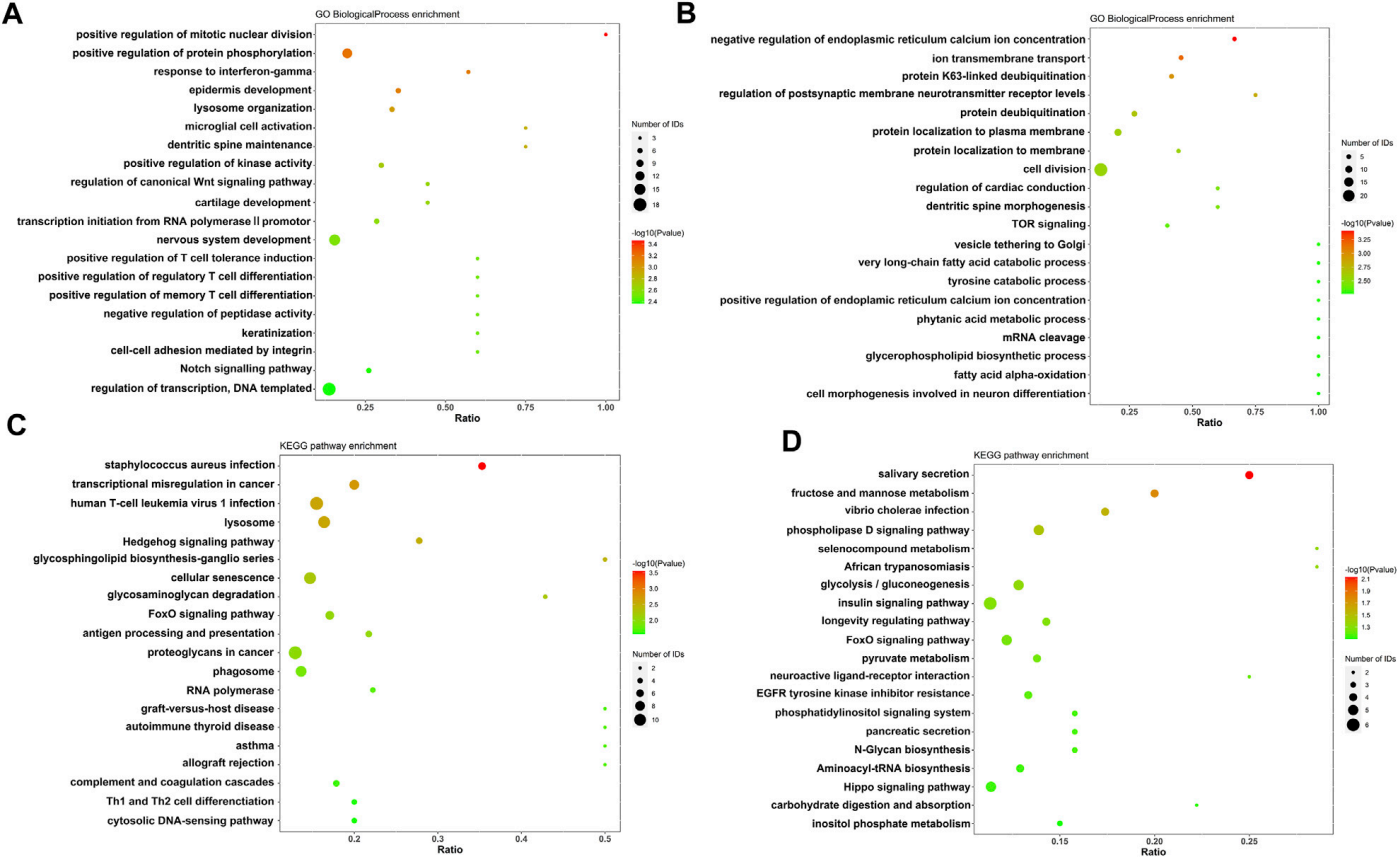
Gene ontology annotation and KEGG pathway analysis of EV-A71-infected RD cells after PZH treatment. **(A)** GO analysis of upregulated proteins. **(B)** GO analysis of downregulated proteins. **(C)** KEGG analysis of upregulated proteins. **(D)** KEGG analysis of downregulated proteins.

Label-free quantitative proteomics was performed to analysis the changes of protein expression between control group and PZH treated group after viral infection. Proteins that exhibited a fold change >1.5 and a statistical significance of *p* < 0.05 were classified as differentially expressed proteins. Moreover, GO and KEGG enrichment was conducted to investigate the function and related biological pathway of the differentially expressed proteins. The most enriched terms are displayed in Figure 4. Compared to the control group, the PZH-treated group exhibited significant upregulation (*p* ≤ 0.01) of proteins involved in host antiviral innate and adaptive immunity (antigen processing and presentation, response to interferon-gamma, regulatory T cell and memory T-cell differentiation and Th1 and Th2 cell differentiation). This suggested that PZH treatment enhanced the host’s immune response against the viral infection. On the other hand, proteins associated with TOR signaling pathway and phospholipase D signaling pathway were downregulated after intervention with PZH (*p* ≤ 0.01), potentially influencing cellular processes such as growth, proliferation, and metabolism. Overall, the functional and pathway changes observed in the differentially expressed proteins are consistent with the physiological effects of PZH.

### 3.4 The antiviral effects of PZH are related to the PI3K/AKT/mTOR and NF-κB signaling pathways

**FIGURE 5:**
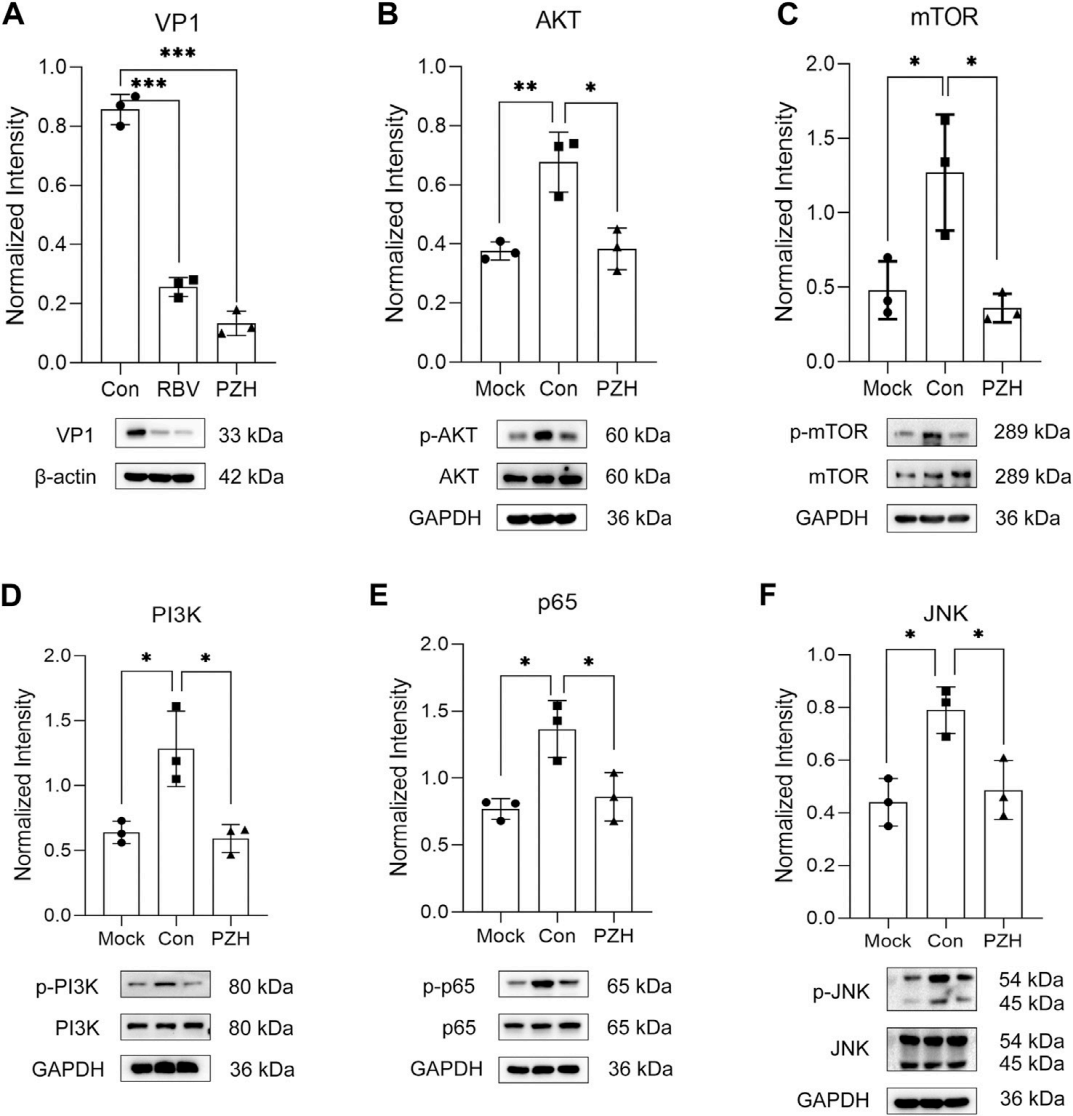
Validation of differently expression proteins. RD cells were mock-infected or infected with EV-A71 (H, MOI = 0.01) for 1 h. Then, cells were subjected to treatment with either PZH or RBV for 24 h. The cells were harvested and the expression of VP1 **(A)**, p-AKT **(B)**, p-mTOR **(C)**, p-PI3K **(D)**, p-p65 **(E)**, p-JNK **(F)** was examined by Western blot. *p < 0.05, **p < 0.01 by *t*-test.

Viral infection can trigger the activation of the PI3K/Akt/mTOR and NF-κB signaling pathways, which benefits viral infection and replication by reducing cell apoptosis, and induces inflammatory cytokines expression (Luo et al., 2017; Bowman et al., 2018). Considering the GO analysis and the regulation of cytokines mentioned earlier, we initially focused on inflammation related pathways. Notably, the TOR signaling proteins were among the top 20 downregulated proteins. Additionally, previous researches have demonstrated that PZH has anti-inflammatory effects through suppressing the NF-κB signaling pathways (Zheng et al., 2019; Lian et al., 2022). Apart from that, studies have shown that mTOR, JNK and NF-κB inhibitors play important roles in reducing EV-A71 replication (Lv et al., 2014; Zhang et al., 2018; Hao et al., 2019). Therefore, we investigated whether these pathways were involved in PZH treatment. As predicted, most of the upregulated proteins (AKT, mTOR, JNK, p65, PI3K) caused by EV-A71 were downregulated after PZH treatment (Figures 5A−F). Some proteins even restored to comparable levels seen in uninfected cells. Based on these findings, we concluded that the inhibition of the PI3K/AKT/mTOR and NF-κB signaling pathways may contribute together to the antiviral activity of PZH. By suppressing these pathways, PZH could potentially hinder viral replication and reduce the inflammatory response triggered by viral infection.

## 4 Discussion

There is a pressing need for safe and effective medicines against enterovirus infection, especially in children, due to the potentially life-threatening cytokine storm and implications associated with EV-A71 infections (Swain et al., 2022). Targeting the host immune response to inhibiting viral replication and cytokine storm represents a viable strategy for the development of anti-EV-A71 drugs. PZH, a traditional Chinese anti-inflammatory drug has been widely used in clinics to treat inflammation-related diseases (Schramm, 1955). However, the network mechanism by which PZH suppresses EV-A71 is not fully elucidated, which limits its extensive clinical application. In recent years, studies on inhibiting viruses by inhibiting the mTOR pathway of host cells have been widely reported (Le Sage et al., 2016; Bowman et al., 2018). Existing mTOR inhibitors have been tested for their activities against EV-A71 *in vitro* (Hao et al., 2019). Furthermore, the importance of PDGFR, PI3K/Akt, p38 MAPK, JNK, and NF-κB in response to EV-A71 infection was also elaborated (Jin et al., 2018). In this study, we first elucidated that PZH could suppress EV-A71 infections through regulating multiple inflammation-related pathways, preliminarily demonstrating its specific antiviral mechanism.

After viral infection, virus can subdue the host cellular response by modulating autophagy and inhibiting apoptosis, thereby benefiting viral replication. It hijacked PI3K/Akt/mTOR signaling pathway to facilitate this process (Le Sage et al., 2016). The mTOR inhibitors have demonstrated efficacy against many viruses such as human immunodeficiency virus (HIV) (Heredia et al., 2015), influenza A virus (Liu et al., 2016), zika virus (Liang et al., 2016) and gamma herpe virus (Pinelli et al., 2015). These inhibitors have also shown potent activities against EV-A71 (Hao et al., 2019). Proteomics analysis and changes in protein expression levels both indicated that PZH could significantly inhibit mTOR signaling pathway, therefore exerting a specific antiviral effect on EV-A71.

In addition, as a multi-component drug, PZH provided robust protection against EV-A71 infection both *in vivo* and *in vitro* by exerting comprehensive regulatory effects on cytokines in plasma and inflammation-related pathways caused by EV-A71. This is particularly vital in the treatment of EV-A71 infection. PZH significantly increased the survival rate of immature mice, decreased the yields of EV-A71 in muscle tissue and signally alleviated clinical manifestations of infected mice, suggesting its potential as an anti-EV-A71 drug for children. Cytokine storm in plasma and central nervous system is one of the most dangerous symptoms closely related to EV-A71 infection and can be fatal (Ye et al., 2015; Swain et al., 2022). The increased secretions of IL-1β, IL-2, TNF-α, IL-13 and KC can be significantly reduced after PZH treatment. Moreover, the upregulated cytokines have been reported to be closely related to the activation of NF-κB and PI3K/Akt/mTOR signaling induced by EV-A71 (Jin et al., 2018; Zhang et al., 2018). Thus, it suggested that the downregulation of these pathways may partially explain the mechanism of protective effects from PZH.

The investigation regarding the potential synergistic antiviral effect of PZH as a multi-component drug, specifically in terms of the contributions of its individual components, remains an ongoing subject of active research.

## 5 Conclusion

Overall, we demonstrated that PZH exerted a good antiviral and protective efficacy on immature mouse model of EV-A71 in a dose-dependent manner and regulated cytokine in serum for the first time. The downregulation of mTOR signaling proteins may be important for PZH exerting both anti-EV-A71 and anti-inflammatory effects, together with the inhibition of other EV-A71-related pathways including PI3K, AKT, JNK and NF-κB signaling pathways. Given that PZH is a commercially available medication, this study holds the potential to expand its clinical indications, and provides a basis for further investigations into the therapeutic potential of PZH for EV-A71 infection.

## Data Availability

The datasets presented in this study can be found in online repositories. The names of the repository/repositories and accession number(s) can be found below: http://www.proteomexchange.org/, PXD042840.
