# Supplementary material for: Evaluation and mechanism study of Pien Tze Huang against EV-A71 infection
Source: Front Pharmacol. 2023 Oct 26;14:1251731. doi: 10.3389/fphar.2023.1251731 (PMC10637388; doi:10.3389/fphar.2023.1251731)
Supplement: Supplementary file 2 [file DataSheet1.docx]

Supplementary Material

# Supplementary Figures and Tables


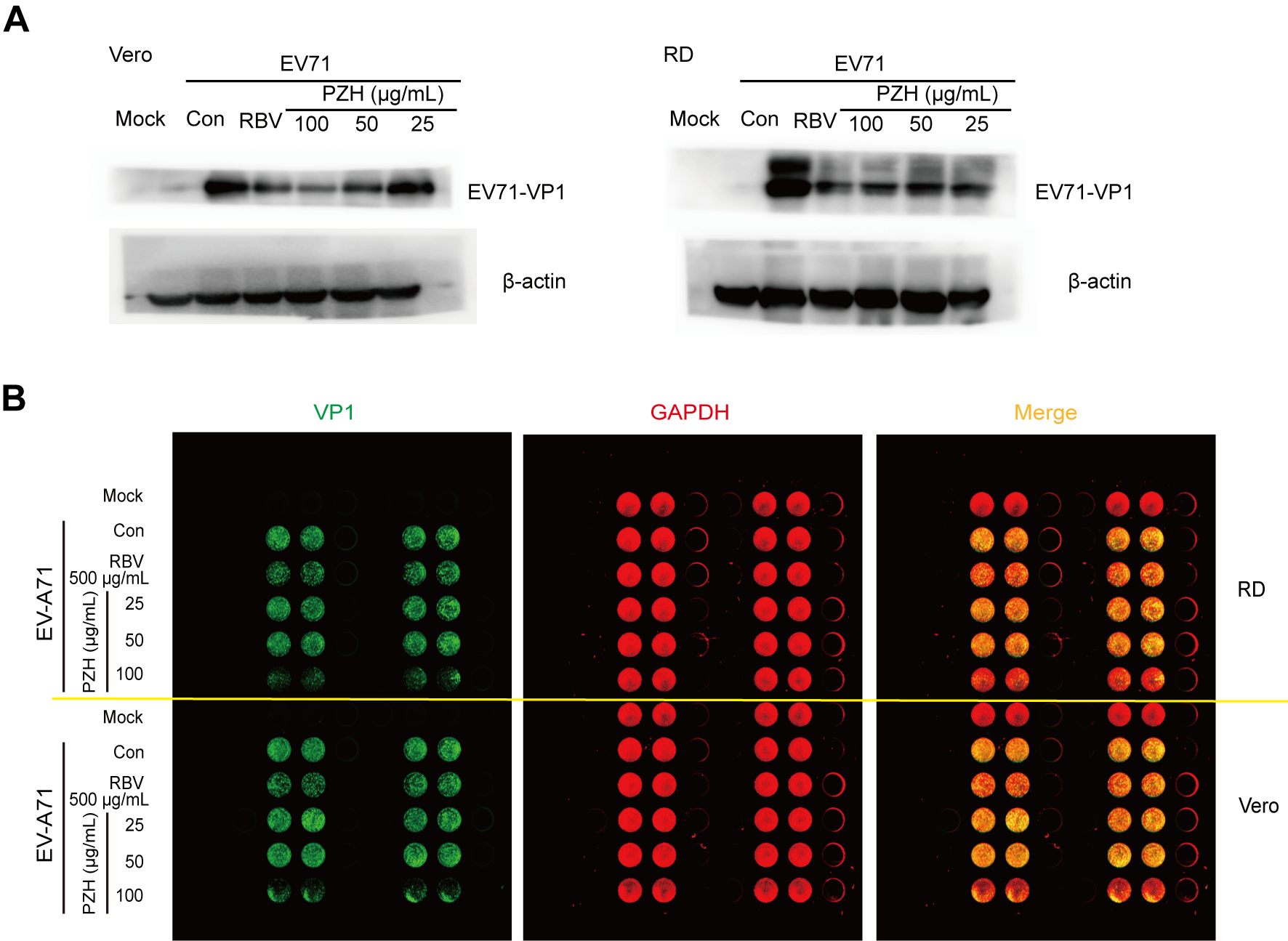


**Fig. S1** Uncropped images of the western blot assay results and In-cell WB assay results shown in Fig. 1A and Fig. 1B.

**Table S1** qRT-PCR assay results shown in Fig. 1C.

|  | **Con** | **100 μg/ml** | **50 μg/ml** | **25** **μg/ml** | **RBV 500μg/ml** |
| --- | --- | --- | --- | --- | --- |
| **Vero (-log10TCID50)** | 1 | 0.18 | 0.4 | 0.59 | 0.23 |
|  | 1 | 0.18 | 0.35 | 0.51 | 0.38 |
|  | 1 | 0.29 | 0.62 | 0.67 | 0.66 |
| **RD (-log10TCID50)** | 1 | 0.22 | 0.3 | 0.53 | 0.37 |
|  | 1 | 0.19 | 0.4 | 0.62 | 0.45 |
|  | 1 | 0.42 | 0.61 | 0.9 | 0.64 |

**Table S2** Viral titers detection by the end-point dilution assay in Vero cells results shown in Fig. 1D.

|  | **Con** | **100 μg/ml** | **50 μg/ml** | **25 μg/ml** | **RBV 500μg/ml** |
| --- | --- | --- | --- | --- | --- |
| **Vero** **(-log10TCID50)** | 5 | 4 | 4 | 4.67 | 4 |
|  | 5.5 | 3.5 | 4.23 | 4.5 | 4 |
|  | 5 | 3 | 4.5 | 5 | 4.23 |
| **RD (-log10TCID50)** | 6 | 5 | 5.5 | 6.5 | 4.5 |
|  | 6.44 | 4.5 | 5 | 6.5 | 5 |
|  | 6.25 | 5 | 5.33 | 6 | 5.5 |

**Table S3** All mice survival results shown in Fig. 2A and Fig. 2B. (0 means live and 1 means death)

| **Day** | **Normal** | **Vehicle** | **RBV 50mg/kg** | **PZH 170 mg/kg** | **PZH 56.7 mg/kg** | **PZH 18.9 mg/kg** |
| --- | --- | --- | --- | --- | --- | --- |
| 2 |  |  |  |  |  |  |
| 5 |  |  | 1 |  |  |  |
| 5 |  |  | 1 |  |  |  |
| 5 |  |  | 1 |  |  |  |
| 6 |  |  | 1 |  |  | 1 |
| 7 |  |  |  | 1 |  |  |
| 8 |  |  |  | 1 | 1 | 1 |
| 8 |  |  |  | 1 | 1 | 1 |
| 8 |  |  |  | 1 |  | 1 |
| 8 |  |  |  | 1 |  |  |
| 8 |  |  |  | 1 |  |  |
| 9 |  |  | 1 |  | 1 |  |
| 9 |  |  | 1 |  |  |  |
| 9 |  |  | 1 |  |  |  |
| 14 | 0 | 0 | 0 | 0 | 0 | 0 |
| 14 | 0 | 0 | 0 | 0 | 0 | 0 |
| 14 | 0 | 0 | 0 | 0 | 0 | 0 |
| 14 | 0 |  | 0 | 0 | 0 | 0 |
| 14 | 0 |  |  | 0 | 0 | 0 |
| 14 | 0 |  |  | 0 | 0 | 0 |
| 14 | 0 |  |  | 0 | 0 | 0 |
| 14 | 0 |  |  | 0 |  |  |
| 14 | 0 |  |  | 0 |  |  |
| 14 | 0 |  |  | 0 |  |  |

**Table S4** All mice clinical scores shown in Fig. 2C.

| **Day** | **Lesion grade** | | | | |
| --- | --- | --- | --- | --- | --- |
| **1** | 0 | 0 | 0 | 0 | 0 |
| **2** | 0 | 0 | 0 | 0 | 0 |
| **3** | 0 | 0 | 0 | 0 | 0 |
| **4** | 0 | 0 | 0 | 0 | 0 |
| **5** | 2 | 2 | 2 | 2 | 1 |
| **6** | 3 | 3 | 2 | 2 | 2 |
| **7** | 4 | 3 | 3 | 3 | 2 |
| **8** | 4 | 3 | 2 | 2 | 2 |
| **9** | 4 | 4 | 4 | 4 | 2 |
| **10** | 4 | 4 | 4 | 4 | 2 |
| **11** | 4 | 4 | 4 | 4 | 2 |
| **12** | 4 | 4 | 4 | 4 | 1 |
| **13** | 4 | 4 | 4 | 4 | 1 |
| **14** | 4 | 4 | 4 | 4 | 0 |

**Table S5** All viral titer assays results of muscle tissues from mice shown in Fig. 2D.

| **EV-A71 Titer (-log_10_TCID_50_)** | **Normal** | **Vehicle** | **RBV 50mg/kg** | **PZH 170 mg/kg** | **PZH 56.7 mg/kg** | **PZH 18.9 mg/kg** |
| --- | --- | --- | --- | --- | --- | --- |
|  | 0 | 4 | 0.5 | 2.67 | 3.2 | 2.83 |
|  | 0 | 3.2 | 3.2 | 2.33 | 3.2 | 3.5 |
|  | 0 | 3.44 | 3.44 | 0.67 | 2.67 | 3.67 |
|  | 0 | 3.2 | 2.67 | 0.8 | 1.33 | 3.92 |
|  | 0 | 3.5 | 2.67 | 1 | 2 | 3.77 |

**Table S6** All IHC analyses of muscle tissues from mice shown in Fig. 2E.

| **Density mean** | **Normal** | **Vehicle** | **RBV 50mg/kg** | **PZH 170 mg/kg** | **PZH 56.7 mg/kg** | **PZH 18.9 mg/kg** |
| --- | --- | --- | --- | --- | --- | --- |
|  | 0.0747 | 0.414133 | 0.1874 | 0.076433 | 0.3535 | 0.268233 |
|  | 0.067733 | 0.4679 | 0.252033 | 0.0416 | 0.287367 | 0.3843 |
|  | 0.0258 | 0.434367 | 0.2406 | 0.246833 | 0.1703 | 0.515133 |
|  | 0.038633 | 0.4113 | 0.2443 | 0.108833 | 0.283 | 0.417733 |
|  | 0.054367 | 0.432533 | 0.2757 | 0.3317 | 0.483867 | 0.167167 |

**Table S7** All H&E stain results of muscle tissues from mice shown in Fig. 2F.

| **Muscular pathlogy score** | **Normal** | **Vehicle** | **RBV 50mg/kg** | **PZH 170 mg/kg** | **PZH 56.7 mg/kg** | **PZH 18.9 mg/kg** |
| --- | --- | --- | --- | --- | --- | --- |
|  | 0 | 4 | 2 | 1 | 1 | 2 |
|  | 0 | 3 | 1 | 0 | 2 | 1 |
|  | 0 | 3 | 1 | 1 | 0 | 2 |
|  | 0 | 4 | 1 | 0 | 3 | 2 |
|  | 0 | 3 | 1 | 2 | 3 | 2 |

**Table S8** All changes of cytokines in mice serum shown in Fig. 3.

| **(pg/ml)** | **Normal** | **Vehicle** | **RBV 50mg/kg** | **PZH 170 mg/kg** | **PZH 56.7 mg/kg** | **PZH 18.9 mg/kg** |
| --- | --- | --- | --- | --- | --- | --- |
|  | 112 | 342 | 331 | 320 | 297 | 287 |
|  | 96 | 451 | 261 | 271.5 | 270 | 324 |
|  | 118 | 742 | 255.5 | 180.5 | 374.5 | 364 |
|  | 157 | 398 | 332 | 235 | 438 | 297 |
|  | 132 | 332 | 283 | 440 | 499 | 360 |

**Table S9** Normalized intensity of western blot for all replicates of differently expression proteins in Fig. 5.

|  | **Mock** | **Con** | **PZH** |
| --- | --- | --- | --- |
| **AKT** | 0.37 | 0.56 | 0.31 |
|  | 0.35 | 0.73 | 0.45 |
|  | 0.41 | 0.74 | 0.39 |
| **mTOR** | 0.41 | 0.85 | 0.32 |
|  | 0.70 | 1.34 | 0.47 |
|  | 0.33 | 1.62 | 0.29 |
| **PI3K** | 0.63 | 1.19 | 0.66 |
|  | 0.73 | 1.61 | 0.47 |
|  | 0.56 | 1.05 | 0.65 |
| **p65** | 0.81 | 1.43 | 0.86 |
|  | 0.82 | 1.54 | 1.04 |
|  | 0.68 | 1.13 | 0.68 |
| **JNK** | 0.40 | 0.83 | 0.65 |
|  | 0.49 | 0.78 | 0.52 |
|  | 0.62 | 0.94 | 0.70 |

**Table S10** Normalized intensity of western blot for all replicates of VP-1 protein in Fig. 5.

|  | **Con** | **RBV** | **PZH** |
| --- | --- | --- | --- |
| **VP-1** | 0.80 | 0.27 | 0.18 |
|  | 0.90 | 0.22 | 0.10 |
|  | 0.87 | 0.28 | 0.12 |


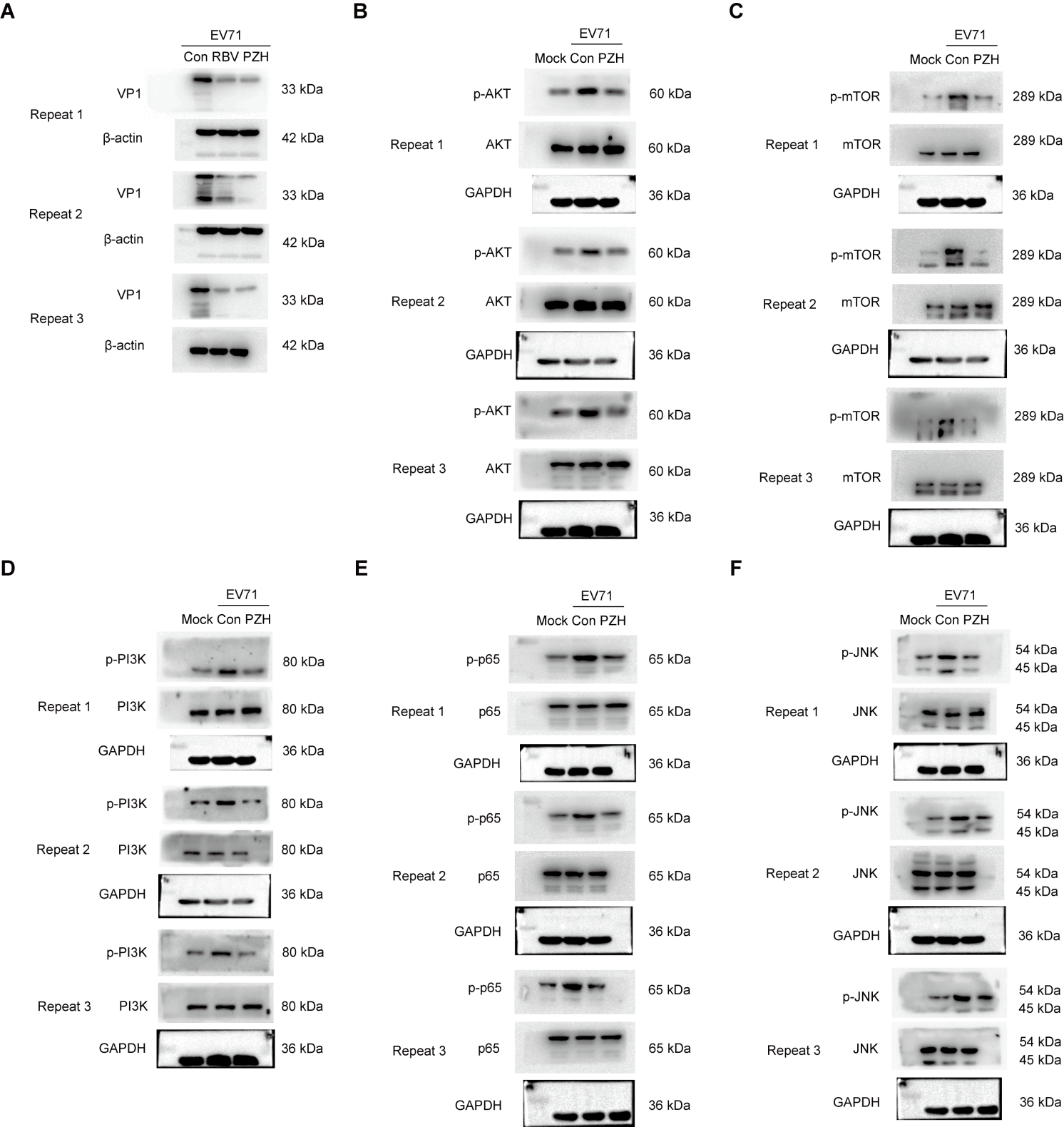


**Fig. S2** Uncropped images of western blot for all replicates of differently expression proteins including VP1 (A), P-AKT (B), P-mTOR (C), P-PI3K (D), P-p65 (E), P-JNK (F) shown in Fig. 5.

# Supplementary Materials of Proteomics Analysis

The supplementary materials of proteomics analysis are in the attachments. The identified proteins are shown in Table S9. Additionally, the GO analysis of up-regulated proteins and down-regulated proteins are shown in Table S10 and Table S11, while the KEGG analysis of up-regulated proteins and down-regulated proteins are shown in Table S12 and Table S13.
